# Evaluation of Each Three *Entamoeba histolytica*- and *Strongyloides stercoralis*-Specific Real-Time PCR Assays Applying Test Comparisons Without Reference Standards

**DOI:** 10.3390/microorganisms13091976

**Published:** 2025-08-24

**Authors:** Andreas Erich Zautner, Hagen Frickmann, Andreas Hahn, Fred Stephen Sarfo, Betty Roberta Norman, Albert Dompreh, Martin Kofi Agyei, Shadrack Osei Asibey, Richard Boateng, Edmund Osei Kuffour, Veronica Di Cristanziano, Tafese Beyene Tufa, Torsten Feldt, Kirsten Alexandra Eberhardt

**Affiliations:** 1 Institute of Medical Microbiology and Hospital Hygiene, Medical Faculty, Otto-von-Guericke University Magdeburg, 39120 Magdeburg, Germany; 2 Department of Microbiology and Hospital Hygiene, Bundeswehr Hospital Hamburg, 22049 Hamburg, Germany; frickmann@bnitm.de; 3 Institute for Medical Microbiology, Virology and Hygiene, University Medicine Rostock, 18057 Rostock, Germany; andreas.hahn@uni-rostock.de; 4 Department of Medicine, Komfo Anokye Teaching Hospital, Kumasi 00233, Ghana; stephensarfo78@gmail.com (F.S.S.); branorman@yahoo.com (B.R.N.); martinagyei@yahoo.co.uk (M.K.A.); shakosbey19@gmail.com (S.O.A.); 5 Kwame Nkrumah University of Science and Technology, Kumasi 00233, Ghana; 6 Department of Clinical Microbiology, Komfo Anokye Teaching Hospital, Kumasi 00233, Ghana; adompreh@gmail.com (A.D.); richardboateng166@gmail.com (R.B.); 7 Laboratory of Retrovirology, The Rockefeller University, New York, NY 10065, USA; eosei@rockefeller.edu; 8 Institute of Virology, Faculty of Medicine and University Hospital Cologne, University of Cologne, 50937 Cologne, Germany; veronica.di-cristanziano@uk-koeln.de; 9 Asella Teaching and Referral Hospital, College of Health Sciences, Arsi University, Asella P.O. Box 04, Ethiopia; tafeseb.tufa@yahoo.com; 10 Hirsch Institute of Tropical Medicine (HITM), Heinrich-Heine University, Asella P.O. Box 04, Ethiopia; torsten.feldt@med.uni-duesseldorf.de; 11 Department of Gastroenterology, Hepatology and Infectious Diseases, Medical Faculty and University Hospital Düsseldorf, Heinrich Heine University Düsseldorf, 40225 Düsseldorf, Germany; 12 Department of Tropical Medicine, Bernhard Nocht Institute for Tropical Medicine & I. Department of Medicine, University Medical Center Hamburg-Eppendorf, 20359 Hamburg, Germany

**Keywords:** *Entamoeba histolytica*, *Strongyloides stercoralis*, real-time PCR, test comparison, latent class analysis

## Abstract

Molecular diagnoses of *Entamoeba histolytica* and *Strongyloides stercoralis* in human samples are becoming increasingly common. To contribute to the ongoing standardization of molecular diagnostic approaches targeting these parasites, we compared three published *E. histolytica*- and *S. stercoralis*-specific real-time PCR assays in test comparisons without a reference standard. Latent class analysis (LCA) was used to calculate diagnostic accuracy estimations for the three compared assays per parameter. The comparison was conducted using stool samples from Ghanaian individuals. In the course of the assessment of 873 stool samples, the number of detected positive PCR results ranged from 10 to 15 for *S. stercoralis* and from 4 to 54 for *E. histolytica* depending on the applied assay. Diagnostic accuracy estimates of real-time PCR sensitivity for *S. stercoralis* and *E. histolytica* ranged from 89% to 100% and from 75% to 100%, respectively; diagnostic estimates of specificity ranged from 99% to 100% and from 94% to 100%, respectively. Diagnostic accuracy-adjusted prevalence estimates were 1.2% for *S. stercoralis* and 0.5% for *E. histolytica*. High cycle threshold values of real-time PCR > 35 showed a particularly reduced likeliness of reproducibility when applying competitor real-time PCR assays. There were no clear-cut differences in terms of diagnostic accuracy favoring either small-subunit ribosomal ribonucleic acid (SSU rRNA) gene sequences or the *S. stercoralis* dispersed repetitive sequence for *S. stercoralis* PCR. The same applied to the comparison of real-time PCRs targeting SSU rRNA gene sequences and the SSU rRNA episomal repeat sequence (SREPH) of *E. histolytica*. In conclusion, interchangeability of the compared real-time PCR assays was higher for the assessed *S. stercoralis* assays compared with the assessed *E. histolytica* assays. Regional diagnostic accuracy testing seems advisable before literature-adapted assays for rare tropical pathogens like *S. stercoralis* and *E. histolytica* are applied in different study regions.

## 1. Introduction

Considering the severity of human enteric infections with eukaryotic parasites, the protozoon *Entamoeba histolytica* and the helminth *Strongyloides stercoralis* are among the clinically most relevant infectious microorganisms as detailed in the following. Accordingly, they should not be missed by the applied diagnostic approaches in areas of endemicity.

In short, *E. histolytica* is the causative agent of human amebiasis [1]. It causes fecal–orally transmitted enteric infections and sometimes even systemic infections associated with poor hygiene conditions. In addition to infections in individuals from usually resource-limited areas of endemicity with restricted access to sanitation and clean water, enteric amebiases has also been reported from travelers, immigrants and individuals practicing anal–oral-sexual intercourse [1]. Diarrhea and dysentery are the most commonly observed manifestations, while systemic spread is most frequently associated with amebic liver abscess. Rarer manifestations include pleuropulmonary or pericardial amebiasis [2]. Enteric microbiome composition and malnutrition affect the severity of the clinical course [3,4]. Deep ulcerations have been associated with enteric amebiasis [5]. Notably, confusion with inflammatory bowel disease and even death can occur [6]. Abscess rupture and thromboses of the hepatic veins and inferior vena cava (with the risk of pulmonary emboli) have been reported as complications of amebic liver abscess [7].

*S. stercoralis* is a soil-transmitted nematode that causes both enteric and systemic, usually chronic infections in human individuals depending on their immunocompetence, culminating in hyperinfection syndrome caused by autoinfecting larvae with larval dissemination into various organs in cases of severe immunosuppression [8]. Unexplained Gram-negative meningitis was identified early as a warning sign for disseminated strongyloidiasis in immunocompromised individuals, as migrating larvae may transport coliform bacteria to the central nervous system [9]. Immunosuppression associated with acquired immunodeficiency syndrome (AIDS), organ transplantation, human T-cell lymphotropic virus 1 (HTLV-1), and even alcoholism has been identified as a risk factor for systemic strongyloidiasis with high mortality risk [10,11]. Infrequently, hyperinfection events have also been described in immunocompetent patients with strongyloidiasis [12]. Common symptoms of strongyloidiasis comprise abdominal pain, diarrhea, vomiting, fever and rash; asymptomatic infections are frequent [13,14]. Eosinophilia is observed in about 70% of patients with strongyloidiasis and may persist after treatment [15]. Next to people living in tropical high endemicity settings [16,17,18,19,20], migrants [13,21,22], travelers, as well as deployed soldiers and policemen are among the individuals at risk [13,23]. The helminth favors humid, wet climates in the subtropics or tropics, where optimum conditions for its free-living cycle exist [24,25], and prevalence is particularly high in warm, moist regions if inadequate sanitary conditions support the nematode’s spread [24,26]. As a public health approach for high-endemicity settings, encouraging progress has been made with ivermectin-based preventive chemotherapy applied to reduce the local prevalence of strongyloidiasis [27], but specific diagnosis is desirable for a targeted therapy with ivermectin or benzimidazoles [28]. Therapy is not only associated with inconsistent cure rates but also with considerable side effects [28].

Focusing on diagnostic aspects, diagnosis of enteric amebiasis is challenged by the fact that *E. histolytica* cysts in human stool samples are microscopically indistinguishable from cysts of apathogenic, merely enterically colonizing amebae like *Entamoeba dispar* [29]. Accordingly, the diagnosis should not rely on microscopy alone but should be confirmed by other diagnostic approaches. While various strategies for confirmation testing, including traditional approaches like antigen-assays [30], molecular staining techniques like fluorescence-in situ-hybridization [31], and special molecular approaches like loop-mediated isothermal amplification (LAMP) [32], recombinase-polymerase-amplification [33] and sequencing [34], have been considered and evaluated so far, polymerase chain reaction (PCR-)-based diagnosis is commonly preferred in times of increasing availability of this approach. Considering a broad variety of published studies on *E. histolytica*-specific real-time PCRs [32,35,36,37,38,39,40,41,42,43,44,45,46,47,48,49,50,51,52], the approach is now considered as well established and quality controlled by external laboratory control schemes [53]. Of note, various authors used the same oligonucleotides in their assessments, and most *E. histolytica*-specific real-time assays amplify three different target sequences, comprising sequence fragments of the small-subunit RNA (ribosomal nucleic acid) gene as well as its episomal repeat sequence [43,48]. These sequences are considered suitable for diagnostic PCRs targeting human amebiasis with sufficient sensitivity and specificity. The differential influence of various pre-analytical factors, including effects of storage and transport media like SAF (sodium acetate glacial acetic acid formalin) [54,55], different modes of nucleic acid extraction [56] and freezing prior to nucleic acid extraction [57], on the diagnostic accuracy of *E. histolytica*-specific real-time PCR, was assessed. Commercial real-time PCR assays, however, frequently operate with fresh stool samples and standard nucleic acid extraction methods with yet satisfying diagnostic reliability [58,59].

Parasitological diagnostic approaches for strongyloidiasis are based on traditional microscopic techniques like direct saline stool microscopy and the Kato–Katz technique, as well as on microscopy with prior enrichment techniques like formol-ether concentration and Baermann–Moraes enrichment next to Koga agar, Dancescu or charcoal culture for the detection of larvae in stool samples; however, low sensitivity remains a major limitation, carrying the risk of overlooking infections [8,18,19,60]. Of note, sensitivity of traditional microscopic parasitology can be increased by the assessment of subsequently sampled specimens over several days [24]. The diagnostic accuracy of serological approaches targeting both helminth antigens and *S. stercoralis*-specific antibodies, in contrast, is limited by considerable cross-reactivity [60,61,62,63], e.g., with antibodies against hookworms, filariae and schistosomes [63], although promising improvements in terms of specificity have recently been made as detailed elsewhere [64,65,66,67]. In cases of successful therapy, *S. stercoralis*-specific antibody titers decline in most patients after one or two years [68]. Modern molecular diagnostic approaches including real-time polymerase chain reaction (PCR), digital droplet PCR (ddPCR) and loop-mediated isothermal amplification (LAMP) have considerably increased diagnostic accuracy in terms of sensitivity and specificity [8,69,70], although sensitivity is not necessarily higher compared to enrichment-based parasitological examinations as indicated by a meta-analysis [70]. Further, lacking standardization in terms of assay protocols considering regional sequence variation of helminth DNA and lacking standardization of sample preparation are ongoing obstacles for molecular diagnostic approaches [8]. Intermittent larval shedding remains another unresolved challenge [60]. Combined diagnostic approaches comprising traditional microbiology, molecular diagnostic assays and serology are considered to provide optimized diagnostic accuracy [19,71]. The value of next-generation sequencing (NGS) techniques for the diagnosis of strongyloidiasis is under investigation as well [60]. Further, restriction fragment length polymorphism (RFLP) analysis of PCR amplicons can be applied to discriminate *S. stercoralis* lineages from human patients and dogs, as well as *Strongyloides fuelleborni* infecting monkeys and *Strongyloides ratti* infecting rodents [64]. As an element of ongoing standardization of the molecular diagnosis of *S. stercoralis* infections, the issue of yet partly dissatisfying sensitivity [70] needs to be addressed. As previously shown for the molecular diagnosis of schistosomiasis, the use of highly repetitive nucleic acid sequences as real-time PCR targets is a promising approach in order to achieve increased sensitivity compared with the use of ribosomal target sequences [72], which have been traditionally applied as target sequences for *S. stercoralis*-specific real-time PCR [73,74,75]. Notably, choosing highly repetitive nucleic acid sequences as real-time PCR targets does not necessarily also increase specificity. Recently, investigators applied NGS to identify highly repetitive sequences within the genome of *S. stercoralis* as promising new PCR targets [75]. Indeed, targeting the so-called *S. stercoralis* dispersed repetitive sequence resulted in an increased number of positive real-time PCR signals compared with traditional real-time PCR targeting the helminth’s small-subunit ribosomal RNA (SSU rRNA) gene [75].

Our study was conducted to comparatively investigate the suitability of the abovementioned sequences for *E. histolytica*-specific real-time PCR [43,48] and to further assess the diagnostic accuracy of real-time PCR targeting the *S. stercoralis* dispersed repetitive sequence [75] when applied with human stool samples. Due to the lack of “gold standards” for the diagnosis of enteric amebiasis and active strongyloidiasis, i.e., assays with 100% sensitivity and specificity each, because of the abovementioned methodical challenges, head-to-head test comparisons without a gold standard were applied based on latent class analysis (LCA) [76], with three real-time PCR assays each targeting sequences of *E. histolytica* and of *S. stercoralis* as competitors in order to provide diagnostic accuracy estimates. In line with the experience from previous works suggesting increased strongyloidiasis prevalence in immunocompromised individuals [77], as well as a pooled prevalence of 2.7% strongyloidiasis in sub-Saharan Africa [19], stool samples from a cohort of Ghanaian individuals for which previous assessments had suggested a considerable rate of strongyloidiasis and enteric amebiasis [78] were used for the assessments. Generally, Ghanaian individuals show high rates of infection or colonization with enteric amebae, especially in remote areas [79]. However, *E. dispar* colonization rates are usually higher than *E. histolytica* infection rates in Ghana [80]. Choosing this approach, the study was intended to contribute to available information on the diagnostic accuracy of *E. histolytica*-specific and *S. stercoralis*-specific real-time PCR from human stool samples.

## 2. Materials and Methods

### 2.1. Ethics

The Declaration of Helsinki and all its amendments were followed for this study. Ethical clearance was granted by the medical association of Hamburg, Germany, (reference number: WF-011/19, obtained on 11 March 2019) for anonymous use of residual sample materials for test comparison purposes without informed consent requirement.

### 2.2. Study Design and Sample Materials

A head-to-head test comparison without a gold standard was chosen as the study design. For the comparative testing, extracted nucleic acids of stool samples collected from 1095 Ghanaian HIV (human immunodeficiency virus) patients as well as from 107 individuals without a known HIV infection [81,82] were applied. From the total number of 1202 individuals originally assessed [81,82] at the study site, 906 sufficiently sized sample materials for the molecular assessments were available.

### 2.3. Laboratory Diagnostics

Prior to the described analyses, all remaining nucleic acid extracts of the assessed Ghanaian stool samples were stored deep-frozen at −80 °C. Nucleic acid extraction was performed using the QIAamp stool DNA mini kit (Qiagen, Hilden, Germany) in line with the manufacturer’s instructions. The three *S. stercoralis*-specific real-time PCR assays chosen for the test comparison comprised two assays targeting SSU rRNA gene sequences of *S. stercoralis* [73,74] as competitors (also referred to as *S. stercoralis* PCR 1 and *S. stercoralis* PCR 2 in the following). The third competitor assay targeted the *Strongyloides stercoralis* dispersed repetitive sequence [75] (also referred to as *S. stercoralis* PCR 3 in the following). The two applied SSU rRNA gene assays used different forward primers [73,74] as a consequence of regional sequence variations in the genome of *S. stercoralis*, as mentioned above [8]. In fact, the forward primer suggested by Basuni and colleagues in 2011 [73] for the *S. stercoralis* PCR 1 was found to target a variable sequence element, leading to the introduction of a new forward primer by Llewellyn and colleagues in 2016 [74] for the *S. stercoralis* PCR 2. These authors considered the problem significant enough to utilize a longer amplicon compared to the design by Basuni and colleagues (details in the Appendix A Table A1). All included samples were analyzed with all three competing real-time PCR assays targeting *S. stercoralis* [73,74,75]. Detailed information on the *S. stercoralis*-specific assays and their specific run conditions, which were slightly adapted from the original publications [73,74,75] to be used with RotorGene Q cyclers (Qiagen, Hilden, Germany), are provided in the Appendix A Table A1 and Table A2 below and elsewhere [61]. In particular, reaction chemistry based on the HotStarTaq (Qiagen, Hilden, Germany) master mix was generally used and cycling times were adapted to cycling profiles showing optimized amplification curves at the used RotorGene Q cyclers. For the *E. histolytica* test comparisons, the same comparative testing was applied with the chosen assays [43,48]. Details on the applied assays and their run conditions, which were also adapted to be used with RotorGene Q cyclers (Qiagen, Hilden, Germany) as described above, are provided in the Appendix A Table A3 and Table A4.

For our assessment, cycle threshold (Ct) values were considered as indicators of target-specific amplification over the whole amplification range as long as typical sigmoid-shaped amplification curves were recorded. As a standard quality control procedure, each real-time PCR run included a plasmid-based positive control (sequence inserts in a pEX A128 vector backbone (eurofins Genomics, Luxemburg)) and a PCR-grade water-based negative control. The Appendix A Table A1 and Table A3 provide sequence details on the positive control sequence inserts as well as on amplicon lengths. The internet-based software “Copy number calculator for realtime PCR” [83] was applied to calculate the technical detection thresholds for each real-time PCR applying ten-fold dilution steps of the positive control plasmids (Appendix A Table A1 and Table A3). A Phocid herpes virus DNA-targeting real-time PCR adapted from the literature [84] was used as an extraction and inhibition control of each sample. Only non-inhibited samples were included in the assessment.

### 2.4. Statistics

Latent class analysis (LCA) [76] was used to calculate diagnostic accuracy estimations of the assays as well as diagnostic accuracy-adjusted prevalence within the assessed study population. LCA is a variant of structural equation models, aiming to estimate latent non-observed variables as the disease status over observed variables, which are the recorded diagnostic test results in this case [76]. Agreement between the diagnostic results as obtained with the compared real-time PCR assays was provided as Fleiss’ kappa [85], and the strata poor (below 0.00), slight (0.00–0.20), fair (0.21–0.40), moderate (0.41–0.60), substantial (0.61–0.80) and almost perfect (0.81–1.00) were assigned as defined elsewhere [85].

## 3. Results

### 3.1. Latent Class Analysis-Based Diagnostic Accuracy Assessment and Diagnostic Accuracy Adjusted Prevalences

Applying the case definitions as described in the methods section above, 873 samples were included in the assessments. As detailed in Table 1, a diagnostic accuracy-adjusted prevalence of 1.2% was calculated for *S. stercoralis*. The number of positive results ranged from 10 (PCR 3) to 15 (PCR 1). Calculated sensitivity of the *S. stercoralis* PCRs ranged between 89% (PCRs 2 and 3) and 100% (PCR 1); calculated specificity ranged between 99% (PCR 1) and 100% (PCR 2 and 3). Substantial agreement between the compared *S. stercoralis* PCRs was observed. Details on matching and mismatching results are shown in Table 2.

For *E. histolytica*, diagnostic accuracy-adjustment resulted in the calculation of a prevalence of 0.5% as shown in Table 3. Between 4 (PCRs 2 and 3) and 54 (PCR 1) positive test results were detected, reflecting differences in specificity and/or sensitivity. Calculated sensitivity ranged between 75% (PCRs 1 and 3) and 100% (PCR 2); specificity ranged between 94% (PCR 1) and 100% (PCRs 2 and 3). Agreement between the assays was slight; details on matching and mismatching positive results are shown in Table 4.

### 3.2. Effects of Cycle Threshold Values

As indicated in Table 5, discordantly positive results when comparing two *S. stercoralis*-specific real-time PCRs were associated with higher Ct values and thus lower quantities of target DNA compared to concordantly positive results. This finding was consistently tested as significant for all conducted comparisons with the exemption of the comparative assessments of positive PCR 3 results with concordant and discordant PCR 1 results, a situation in which the low number of discordant results did not allow for statistical assessments. In most situations, positive test results with Ct values > 35 tended to be non-reproducible with competitor assays. PCR 1 was an exemption when compared to PCR 3, a situation in which even lower Ct values showed an increased likelihood of non-reproducibility.

As shown in Table 6, similar trends were observed for the conducted head-to-head comparisons of *E. histolytica*-specific real-time PCRs. The observed overall low reproducibility of positive PCR 1 results when applying the competitor assays, however, was the reason for lacking statistical significance with the exemption of the comparative assessments of positive PCR 1 results with concordant and discordant PCR 3 results. For the compared *E. histolytica*-specific real-time PCRs 2 and 3, the abovementioned trend for positive test results with Ct values > 35 being non-reproducible with competitor assays was confirmed. This was similar as observed for *S. stercoralis*. Only for PCR 1, for which reduced specificity was suggested above, the trend even pointed towards reduced reproducibility with competitor assays in case of Ct values > 30.

## 4. Discussion

The study was conducted to comparatively assess three real-time PCR assays, each for *S. stercoralis* and *E. histolytica*, respectively. It was conducted as a head-to-head test comparison in the absence of a perfect gold standard. Human stool samples from the Ghanaian area of endemicity were applied. The assessment led to a number of results.

For *S. stercoralis*, the diagnostic accuracy-adjusted prevalence estimation of 1.2% was lower than the pooled prevalence of 2.7% strongyloidiasis as described for sub-Saharan Africa in the literature [19]. It may be hypothesized that this lower prevalence estimation may be explained by increased medical care for the assessed HIV cohort. However, the ethical requirement for fully anonymized use of the samples for test comparison purposes did not allow further investigations of this in our study. Focusing on the calculated diagnostic accuracy estimates for the assessed real-time PCR approaches, calculated specificity was in the 99–100% range for all *S. stercoralis*-specific assays. Calculated sensitivity was recorded in the 89% to 100% range. Notably, the reported increased sensitivity of *S. stercoralis*-specific real-time PCR targeting the *S. stercoralis* dispersed repetitive sequence (PCR 3 in this study) in comparison to assays targeting SSU rRNA sequences (PCRs 1 and 2 in this study) [75] was not confirmed according to our results. In contrast, PCR 3 detected one positive signal not detected by PCR 1 and two positive signals not detected by PCR 3, while it failed to confirm six signals detected by PCR 1 and five signals detected by PCR 2. Regarding the low overall prevalence of *S. stercoralis* and some very high Ct values of recorded real-time PCR signals, this finding should not be overinterpreted, but at the very least, it does not confirm a tremendous sensitivity increase associated with the particular PCR target. On the quantitative level, positive signals with high Ct values >35 and associated low quantities of target DNA tended to be poorly reproducible with competitor assays. This phenomenon was recorded for nearly all assessed *S. stercoralis*-specific assays and is insofar not surprising, as detectability of low target DNA amounts close to the detection threshold generally becomes stochastic [86]. Hypothetically, optimization of nucleic acid extraction might have had a more pronounced effect on diagnostic sensitivity than changes in the real-time PCR target sequence as suggested elsewhere [87,88,89]. Unfortunately, the framework of the study, including the requirement of working with already extracted nucleic acid residual samples, did not allow for further elaboration on this. Nevertheless, considering the recorded substantial agreement between the compared *S. stercoralis*-specific real-time PCR assays, they seem widely interchangeable with negligible impact on diagnostic accuracy. Taking even minor differences in sensitivity and specificity estimates into account, PCR 1 targeting the *S. stercoralis* SSU rRNA gene showed the most promising diagnostic accuracy, followed by PCR 2 targeting another SSU rRNA gene sequence and by PCR 3 targeting the *S. stercoralis* dispersed repetitive sequence on a comparable level.

Focusing on the comparison of the diagnostic accuracy estimations of the assessed three *E. histolytica*-specific real-time PCR assays, the estimated reduced specificity of 94% as calculated for PCR 1 came partly unexpected when compared with the results of previous assessments [58,59]. In a recent diagnostic accuracy assessment with samples from travel returnees, this PCR had shown specificity of 99.4% [58]. Notably, however, lower specificity of 97% had been estimated for this assay when applied with human stool samples collected at tropical areas of endemicity [59]. Further, the specificity estimate of 94% as proposed in the study provided here is still within the lower margin of the 95% confidence interval of the specificity estimate of this recent assessment [59]. Insofar, our results once more confirm a need for regional test evaluations depending on the geographic assessment site as proposed elsewhere [86]. This is particularly true for complex sample materials like human stool, because geographical variations in stool microbiome compositions may easily lead to varying abundance of potentially cross-reacting nucleic acid sequences. Considering the fact that the main share of high Ghanaian stool colonization rates with amebae [79] has been shown to be due to non-pathogenic species [80], the accuracy-adjusted prevalence estimation for *E. histolytica* of 0.5% in our study is epidemiological plausible. In contrast, a measured prevalence of >6% would result when assessing the positive signals of PCR 1 alone. This is a consequence of the lower specificity estimate of this assay in comparison with PCR 2 and 3, for which specificity of virtually 100% has been estimated. The seemingly large sensitivity estimate difference range between 75% and 100% is a consequence of the low proportion of assigned true positivity in latent class analysis. This is confirmed by the wide 95% confidence intervals of the sensitivity estimates and the low number of discordant *E. histolytica* real-time PCR results when ignoring the high number of non-confirmed positive results of PCR 1. Similarly like described for the *S. stercoralis* assays above, samples with high Ct values and thus low amounts of *E. histolytica* target DNA were more likely not to be positively detected by competitor assays, although the low proportion of concordant results prevented significant P values. Notably, positive results of PCR 1 tended to be non-reproducible with competitor assays even at lower Ct values and thus higher amounts of target DNA. This speaks in favor of the hypothesis that several positive PCR 1 results were indeed more likely to be due to non-specific reactions rather than to be results of low target DNA quantities close to the PCRs’ diagnostic detection threshold. Taken together, the comparably low specificity estimate of PCR 1 obtained with the assessed sample collection and the merely slight agreement between the compared *E. histolytica* real-time PCR assays suggest that the compared real-time PCR assays are poorly interchangeable when applying them for screening purposes with stool samples of Ghanaian patients. Considering both sensitivity and specificity estimates, the *E. histolytica* SSU rRNA gene-specific PCR 2 scored best, followed by the small-subunit RNA episomal repeat sequence-specific PCR 3 at second place, and PCR 1, which targets a SSU rRNA gene sequence different from the target sequence of PCR 2, only at third place due to its reduced specificity when applied with stool samples from the assessed study cohort. Unfortunately, due to restricted funding options not allowing for sequence-based analysis of likely cross-reaction events, a further characterization of these events on the molecular level was not feasible in this study setting.

This study has a number of limitations. First, the interpretation of the results is limited by the available number of samples for the assessments and the prevalence of the target microorganisms within the included samples. Second, limited resources did not allow for a comparison of different nucleic acid extraction strategies that might potentially influence diagnostic sensitivity, or for sequence-based evaluation of potentially false positive amplicons to verify or falsify specific amplification in terms of assay specificity [56,57,87,88,89]. Thus, diagnostic accuracy estimates had to rely on latent class analysis only. Third, although molecular approaches have been shown to be considerably more sensitive than microscopy for the diagnosis of enteric parasites [58,88], the unavailability of microscopy results is another study limitation. Fourth, the demand of the underlying ethical clearance for completely anonymized use of the residual samples for test comparison purposes did not allow the use of clinical information to estimate the likelihood at least of clinically apparent infection events. This is an admitted deviation from the requirements of the STARD (Standards for Reporting Diagnostic Accuracy) criteria [90].

## 5. Conclusions

In spite of the abovementioned limitations, the study provided insights into the diagnostic accuracy of a number of published real-time PCR assays targeting rare tropical pathogens like *S. stercoralis* and *E. histolytica*. Thus, it can guide the choice of such assays for epidemiological studies, in which the use of literature-adapted in-house approaches is desired for reasons of cost effectiveness due to limited funding resources, precluding the use of well-characterized commercial assays. Further, the work can help to guide the choice of PCR target sequences by test developers addressing the rare tropical parasites *S. stercoralis* and *E. histolytica* as elements of their assays.

## Figures and Tables

**Table 1:** Agreement kappa between the compared real-time screening PCR assays targeting *S. stercoralis* as well as sensitivity, specificity and accuracy-adjusted prevalence as calculated with latent class analysis (LCA).

Assay	N	Positives (%)	Sensitivity (0.95 CI)	Specificity (0.95 CI)	Kappa (0.95 CI)
*Strongyloides stercoralis* PCR 1	873	15 (1.72)	1 (0, 1)	0.99 (0.86, 1)	0.680 (0.475, 0.856)
*Strongyloides stercoralis* PCR 2	873	13 (1.49)	0.89 (0.50, 0.98)	1 (0.88, 1)	
*Strongyloides stercoralis* PCR 3	873	10 (1.15)	0.89 (0.49, 0.99)	1 (0.99, 1)	
Prevalence of *Strongyloides stercoralis*	1.15%				

N = number included after exclusion of inhibited samples. 0.95 CI = 95% confidence interval.

**Table 2:** Cross-table detailing mismatches between the real-time screening PCRs targeting *S. stercoralis*. Green = matching results. Red = mismatching results. Black = not filled in to avoid repetition.

		*Strongyloides Stercoralis* PCR 1		*Strongyloides Stercoralis* PCR 2		*Strongyloides Stercoralis* PCR 3	
		Negative	Positive	Negative	Positive	Negative	Positive
*Strongyloides stercoralis* PCR 1	Negative			854	4	857	1
	Positive			6	9	6	9
*Strongyloides stercoralis* PCR 2	Negative					858	2
	Positive					5	8
*Strongyloides stercoralis* PCR 3	Negative						
	Positive						

**Table 3:** Agreement kappa between the compared real-time screening PCR assays targeting *Entamoeba histolytica* as well as sensitivity, specificity and accuracy-adjusted prevalence as calculated with latent class analysis (LCA).

Assay	N	Positives (%)	Sensitivity (0.95 CI)	Specificity (0.95 CI)	Kappa (0.95 CI)
*Entamoeba histolytica* PCR 1	873	54 (6.19)	0.75 (0.24, 0.97)	0.94 (0.92, 0.96)	0.108 (0.018, 0.313)
*Entamoeba histolytica* PCR 2	873	4 (0.46)	1 (0, 1)	1 (n.e.)	
*Entamoeba histolytica* PCR 3	873	4 (0.46)	0.75 (0.24, 0.97)	1 (0.99, 1)	
Prevalence of *Entamoeba histolytica*	0.46%				

N = number included after exclusion of inhibited samples. n.e. = not estimable. 0.95 CI = 95% confidence interval.

**Table 4:** Cross-table detailing mismatches between the real-time screening PCRs targeting *Entamoeba histolytica*. Green = matching results. Red = mismatching results. Black = not filled in to avoid repetition.

		*Entamoeba Histolytica* PCR 1		*Entamoeba Histolytica* PCR 2		*Entamoeba Histolytica* PCR 3	
		Negative	Positive	Negative	Positive	Negative	Positive
*Entamoeba histolytica* PCR 1	Negative			818	1	817	2
	Positive			51	3	52	2
*Entamoeba histolytica* PCR 2	Negative					868	1
	Positive					1	3
*Entamoeba histolytica* PCR 3	Negative						
	Positive						

**Table 5:** Comparison of recorded Ct values of the real-time screening PCR assays targeting *S. stercoralis* for samples showing concordant and discordant test results.

	n	Mean (SD)	Median (Min., Max.)	Significance P (*t*-Test)
*Strongyloides stercoralis* PCR 1—concordantly positive results with PCR 2	9	25.59 (3.12)	25.19 (23.32, 32.4)	0.0004
*Strongyloides stercoralis* PCR 1—discordantly positive results with PCR 2	6	32.36 (2.02)	32.88 (29.22, 34.88)	
*Strongyloides stercoralis* PCR 1—concordantly positive results with PCR 3	9	27.29 (3.30)	26.17 (23.32, 32.40)	0.0490
*Strongyloides stercoralis* PCR 1—discordantly positive results with PCR 3	6	31.31 (3.83)	32.88 (24.5, 34.88)	
*Strongyloides stercoralis* PCR 2—concordantly positive results with PCR 1	9	33.33 (2.45)	33 (30, 37)	<0.0001
*Strongyloides stercoralis* PCR 2—discordantly positive results with PCR 1	4	42.25 (1.26)	42 (41, 44)	
*Strongyloides stercoralis* PCR 2—concordantly positive results with PCR 3	8	33.5 (2.56)	33.5 (30, 37)	0.0064
*Strongyloides stercoralis* PCR 2—discordantly positive results with PCR 3	5	40.2 (4.71)	42 (32, 44)	
*Strongyloides stercoralis* PCR 3—concordantly positive results with PCR 1	9	31.44 (2.07)	31 (29, 36)	n.e.
*Strongyloides stercoralis* PCR 3—discordantly positive results with PCR 1	1	35 (-)	35 (35, 35)	
*Strongyloides stercoralis* PCR 3—concordantly positive results with PCR 2	8	30.88 (1.25)	31 (29, 33)	0.0012
*Strongyloides stercoralis* PCR 3—discordantly positive results with PCR 2	2	35.5 (0.71)	35.5 (35, 36)	

Min. = minimum. Max. = maximum. SD = standard deviation. n.e. = not estimable.

**Table 6:** Comparison of recorded Ct values of the real-time screening PCR assays targeting *Entamoeba histolytica* for samples showing concordant and discordant test results.

	n	Mean (SD)	Median (Min., Max.)	Significance P (*t*-Test)
*Entamoeba histolytica* PCR 1—concordantly positive results with PCR 2	3	23.75 (9.57)	24.09 (14.01, 33.15)	0.1218
*Entamoeba histolytica* PCR 1—discordantly positive results with PCR 2	51	37.83 (3.71)	38.33 (18.1, 43.97)	
*Entamoeba histolytica* PCR 1—concordantly positive results with PCR 3	2	19.05 (7.13)	19.05 (14.01, 24.09)	<0.0001
*Entamoeba histolytica* PCR 1—discordantly positive results with PCR 3	52	37.74 (3.73)	38.30 (18.1, 43.97)	
*Entamoeba histolytica* PCR 2—concordantly positive results with PCR 1	3	27 (12.12)	29 (14, 38)	n.e.
*Entamoeba histolytica* PCR 2—discordantly positive results with PCR 1	1	21 (-)	21 (21, 21)	
*Entamoeba histolytica* PCR 2—concordantly positive results with PCR 3	3	21.33 (7.51)	21 (14, 29)	n.e.
*Entamoeba histolytica* PCR 2—discordantly positive results with PCR 3	1	38 (-)	38 (38, 38)	
*Entamoeba histolytica* PCR 3—concordantly positive results with PCR 1	2	21 (5.66)	21 (17, 25)	0.5084
*Entamoeba histolytica* PCR 3—discordantly positive results with PCR 1	2	28.5 (12.02)	28.5 (20, 37)	
*Entamoeba histolytica* PCR 3—concordantly positive results with PCR 2	3	20.67 (4.04)	20 (17, 25)	n.e.
*Entamoeba histolytica* PCR 3—discordantly positive results with PCR 2	1	37 (-)	37 (37, 37)	

Min. = minimum. Max. = maximum. SD = standard deviation. n.e. = not estimable.

## Data Availability

The original contributions presented in this study are included in the article. Further inquiries can be directed to the corresponding author.

## Abbreviations

The following abbreviations are used in this manuscript:

0.95 CI	95% confidence interval
AIDS	acquired immunodeficiency syndrome
Ct	cycle threshold
ddPCR	digital droplet polymerase chain reaction
HIV	human immunodeficiency virus
LAMP	loop-mediated isothermal amplification
LCA	latent class analysis
n	number
n.e.	not estimable
NGS	next generation sequencing
max	maximum
min	minimum, minute
PCR	polymerase chain reaction
RNA	ribosomal nucleic acid
SD	standard deviation
sec	second
SREPH	small-subunit RNA episomal repeat sequence
SSU	small subunit

## Appendix A

**Table A1:** Target genes, calculated detection limits and oligonucleotides used for the applied real-time PCR assays targeting *Strongyloides stercoralis*. Hyphens in the oligonucleotide sequences have been inserted to increase the readability, not to delineate codon triplets.

PCR Target	*Strongyloides stercoralis* (PCR 1)
Target gene	Small-subunit rRNA gene
Detection limit	1.3 × 10^2^ copies/µL
Amplicon size	101 base pairs
Forward primer	5′-GAA-TTC-CAA-GTA-AAC-GTA-AGT-CAT-TAG-C-3′
Reverse primer	5′-TGC-CTC-TGG-ATA-TTG-CTC-AGT-TC-3′
Probe and modifications	5′-CY5-ACA-CAC-CGG-CCG-TCG-CTG-C-BHQ2-3′
Positive control plasmid insert	5′-AAC-GAG-GAA-TTC-CAA-GTA-AAC-GTA-AGT-CAT-TAG-CTT-ACA-TTG-ATT-ACG-TCC-CTG-CCC-TTT-GTA-CAC-ACC-GGC-CGT-CGC-TGC-CCG-GAA-CTG-AGC-AAT-ATC-CAG-AGG-CAG-GAA-GA-3′
GenBank accession number used for the insert	AF279916.2
Reference	[73]
PCR target	*Strongyloides stercoralis* (PCR 2)
Target gene	Small-subunit rRNA gene
Amplicon size	471 base pairs
Detection limit	4.0 × 10^1^ copies/µL
Forward primer	5′-GGG-CCG-GAC-ACT-ATA-AGG-AT-3′
Reverse primer	5′-TGC-CTC-TGG-ATA-TTG-CTC-AGT-TC-3′
Probe and modifications	5′-Cy5-ACA-CAC-CGG-CCG-TCG-CTG-C-BHQ2-3′
Positive control plasmid insert	5′-AAA-CTC-ACC-CGG-GCC-GGA-CAC-TAT-AAG-GAT-TGA-CAG-ATT-GAT-AGC-TCT-TTC-ATG-ATT-TAG-TGG-TTG-GTG-GTG-CAT-GGC-CGT-TCT-TAG-TTC-GTG-GAT-ATG-ATT-TGT-CTG-GTT-GAT-TCC-GAT-AAC-GAG-CGA-GAC-TTT-TAT-GTT-ATA-TTA-AAT-ATT-ATT-ATT-TTG-TTT-ATT-TTA-ATA-TAA-ATA-ATT-AAT-ATT-TTA-ATA-ACA-GAT-TAA-TAG-TGT-TTA-ACT-ATT-TGA-GAG-AGA-GCG-ATA-ACA-GGT-CTG-TGA-TGC-CCT-TAG-ATG-TCC-GGG-GCT-GCA-CGC-GCG-CTA-CAA-TGT-AGT-GAT-CAT-TAT-GTT-CCT-GTT-TAG-AGA-TAA-ATG-GGT-AAA-CAT-TGA-AAA-CAT-TAC-GTA-ACT-GGG-AAT-GAA-AAT-TGC-AAT-TAT-TTT-TCA-TGA-ACG-AGG-AAT-TCC-AAG-TAA-ACG-TAA-GTC-ATT-AGC-TTA-CAT-TGA-TTA-CGT-CCC-TGC-CCT-TTG-TAC-ACA-CCG-GCC-GTC-GCT-GCC-CGG-AAC-TGA-GCA-ATA-TCC-AGA-GGC-AGG-AAG-AGA-T-3′
GenBank accession number used for the insert	AF279916.2
Reference	[74]
PCR target	*Strongyloides stercoralis* (PCR 3)
Target gene	*Strongyloides stercoralis* dispersed repetitive sequence
Amplicon size	136 base pairs
Detection limit	7.5 × 10^1^ copies/µL
Forward primer	5′-CGC-TCC-AGA-ATT-AGT-TCC-AGT-T-3′
Reverse primer	5′-GCA-GCT-TAG-TCG-AAA-GCA-TAG-A-3′
Probe and modifications	5′-6-FAM-ACA-GTC-TCC-AGT-TCA-CTC-CAG-AAG-AGT-BMN-Q535-3′
Positive control plasmid insert	5′-ACA-GCT-CTC-ACG-CTC-CAG-AAT-TAG-TTC-CAG-TTG-AAT-AAC-AGT-CTC-CAG-TTC-ACT-CCA-GAA-GAG-TTC-CTA-TAA-TCC-TAA-CTC-AGC-TCC-AGT-AAA-GCA-ACA-GTT-TCC-AAC-CCC-TCA-CAA-AAG-AGC-TTC-TAT-GCT-TTC-GAC-TAA-GCT-GCA-GTA-TAG-GTA-3′
GenBank accession number used for the insert	AY028262.1
Reference	[75]

**Table A2:** Reaction mixes and run conditions for the real-time screening PCR assays targeting *Strongyloides stercoralis*.

	*Strongyloides stercoralis* PCR 1	*Strongyloides stercoralis* PCR 2	*Strongyloides stercoralis* PCR 3
Reaction chemistry			
Master Mix	HotStar (Qiagen, Hilden, Germany)	HotStar (Qiagen, Hilden, Germany)	HotStar (Qiagen, Hilden, Germany)
Reaction volume (µL)	20.0	20.0	20.0
Forward primer concentration (nM)	60.0	100.0	250.0
Reverse primer concentration (nM)	60.0	100.0	250.0
Probe concentration (nM)	250.0	100.0	125.0
Final Mg^2+^ concentration (mM)	5.0	5.0	5.0
Eluate volume (µL)	2.0	2.0	2.0
Run conditions			
Initial denaturation	15 min. at 95 °C	15 min. at 95 °C	15 min. at 95 °C
Cycle numbers	50	45	45
Denaturation	9 s at 95 °C	15 s at 95 °C	15 s at 95 °C
Annealing and amplification	60 s at 60 °C	60 s at 59 °C	60 s at 59 °C
Hold	30 s at 40 °C	20 s at 30 °C	20 s at 30 °C

min. = minute, s = second.

**Table A3:** Target genes, calculated detection limits and oligonucleotides used for the applied real-time PCR assays for *Entamoeba histolytica*. Hyphens in the oligonucleotide sequences have been inserted to increase the readability, not to delineate codon triplets.

PCR Target	*Entamoeba histolytica* (PCR 1)
Target gene	Small-subunit rRNA gene
Detection limit	1.0 × 10^3^ copies/µL
Amplicon size	173 base pairs
Forward primer	5′-ATT-GTC-GTG-GCA-TCC-TAA-CTC-A-3′
Reverse primer	5′-GCG-GAC-GGC-TCA-TTA-TAA-CA-3′
Probe and modifications	5′-JOE-TCA-TTG-AAT-GAA-TTG-GCC-ATT-T-BHQ1-3′
Positive control plasmid insert	5′-GGA-TGA-AAC-TGC-GGA-CGG-CTC-ATT-ATA-ACA-GTA-ATA-GTT-TCT-TTG-GTT-AGT-AAA-ATA-CAA-GGA-TAG-CTT-TGT-GAA-TGA-TAA-AGA-TAA-TAC-TTG-AGA-CGA-TCC-AGT-TTG-TAT-TAG-TAC-AAA-ATG-GCC-AAT-TCA-TTC-AAT-GAA-TTG-AGA-AAT-GAC-ATT-CTA-AGT-GAG-TTA-GGA-TGC-CAC-GAC-AAT-TGT-AGA-ACA-C-3′
GenBank accession number used for the insert	X64142
Reference	[48]
PCR target	*Entamoeba histolytica* (PCR 2)
Target gene	Small-subunit rRNA gene
Amplicon size	108 base pairs
Detection limit	7.0 × 10^0^ copies/µL
Forward primer	5′-GGA-CAC-ATT-TCA-ATT-GTC-CTA-3′
Reverse primer	5′-CAT-CAC-AGA-CCT-GTT-ATT-GCT-G-3′
Probe and modifications	5′-Cy5-TGT-AGT-TAT-CTA-ATT-TCG-GTT-AGA-CC-BHQ2-3′
Positive control plasmid insert	5′-CTT-CTT-AAA-GGG-ACA-CAT-TTC-AAT-TGT-CCT-ATT-TTA-ATT-GTA-GTT-ATC-TAA-TTT-CGG-TTA-GAC-CTC-TTT-TAA-CGT-GGG-AAA-AAG-AAA-AAG-GAA-GCA-TTC-AGC-AAT-AAC-AGG-TCT-GTG-ATG-CCC-TTA-GAC-A-3′
GenBank accession number used for the insert	X64142
Reference	[43]
PCR target	*Entamoeba histolytica* (PCR 3)
Target gene	small-subunit RNA episomal repeat sequence (SREPH)
Amplicon size	83 base pairs
Detection limit	4.6 × 10^1^ copies/µL
Forward primer	5′-CAT-TAA-AAA-TGG-TGA-GGT-TCT-TAG-GAA-3′
Reverse primer	5′-TGG-TCG-TCG-TCT-AGG-CAA-AAT-ATT-3′
Probe and modifications	5′-FAM-TTG-ACC-AAT-TTA-CAC-CGT-TGA-TTT-TCG-BHQ1-3′
Positive control plasmid insert	5′-TAG-TAC-TTT-TCA-TTA-AAA-ATG-GTG-AGG-TTC-TTA-GGA-AAT-CCG-AAA-ATC-AAC-GGT-GTA-AAT-TGG-TCA-AAA-AAT-ATT-TTG-CCT-AGA-CGA-CGA-CCA-TTT-TGA-ATA-A-3′
GenBank accession number used for the insert	no GenBank accession number, sequence from [91]
Reference	[48]

**Table A4:** Reaction mixes and run conditions for the real-time screening PCR assays for *Entamoeba histolytica*.

	*Entamoeba histolytica* PCR 1	*Entamoeba histolytica* PCR 2	*Entamoeba histolytica* PCR 3
Reaction chemistry			
Master Mix	HotStarTaq (Qiagen, Hilden, Germany)	HotStarTaq (Qiagen, Hilden, Germany)	HotStarTaq (Qiagen, Hilden, Germany)
Reaction volume (µL)	20	20	20
Forward primer concentration (pmol/µL)	125	500	500
Reverse primer concentration (pmol/µL)	125	500	500
Probe concentration (pmol/µL)	175	200	200
Final Mg^2+^ concentration (mM)	5.0	1.5	1.5
Bovine serum albumin (mg/mL)	-	2.0	2.0
Eluate volume (µL)	2.0	2.0	2.0
Run conditions			
Initial denaturation	95 °C for 15 min.	95 °C for 15 min.	95 °C for 15 min.
Cycle numbers	45	40	40
Denaturation	95 °C for 15 s	95 °C for 15 s	95 °C for 15 s
Annealing	Touchdown from 72 °C to 67 °C in 0.5 °C steps for 30 s	Touchdown from 60 °C to 52 °C in 0.5 °C steps for 30 s	Touchdown from 60 °C to 52 °C in 0.5 °C steps for 30 s
Amplification	together with annealing	72 °C for 30 s	72 °C for 30 s
Hold	40 °C for 30 s	30 °C for 20 s	30 °C for 20 s

min. = minute, s = second.
